# Roles of HLA-G/KIR2DL4 in Breast Cancer Immune Microenvironment

**DOI:** 10.3389/fimmu.2022.791975

**Published:** 2022-02-03

**Authors:** Guoxu Zheng, Lintao Jia, An-Gang Yang

**Affiliations:** ^1^State Key Laboratory of Cancer Biology, Department of Immunology, Fourth Military Medical University, Xi’an, China; ^2^State Key Laboratory of Cancer Biology, Department of Biochemistry and Molecular Biology, Fourth Military Medical University, Xi’an, China

**Keywords:** HLA-G, KIR2DL4, breast cancer, immune microenvironment, immunotherapy

## Abstract

Human leukocyte antigen (HLA)-G is a nonclassical MHC Class I molecule, which was initially reported as a mediator of immune tolerance when expressed in extravillous trophoblast cells at the maternal-fetal interface. HLA-G is the only known ligand of killer cell immunoglobulin-like receptor 2DL4 (KIR2DL4), an atypical family molecule that is widely expressed on the surface of NK cells. Unlike other KIR receptors, KIR2DL4 contains both an arginine–tyrosine activation motif in its transmembrane region and an immunoreceptor tyrosine-based inhibitory motif (ITIM) in its cytoplasmic tail, suggesting that KIR2DL4 may function as an activating or inhibitory receptor. The immunosuppressive microenvironment exemplified by a rewired cytokine network and upregulated immune checkpoint proteins is a hallmark of advanced and therapy-refractory tumors. Accumulating evidence has shown that HLA-G is an immune checkpoint molecule with specific relevance in cancer immune escape, although the role of HLA-G/KIR2DL4 in antitumor immunity is still uncharacterized. Our previous study had shown that HLA-G was a pivotal mediator of breast cancer resistance to trastuzumab, and blockade of the HLA-G/KIR2DL4 interaction can resensitize breast cancer to trastuzumab treatment. In this review, we aim to summarize and discuss the role of HLA-G/KIR2DL4 in the immune microenvironment of breast cancer. A better understanding of HLA-G is beneficial to identifying novel biomarker(s) for breast cancer, which is important for precision diagnosis and prognostic assessment. In addition, it is also necessary to unravel the mechanisms underlying HLA-G/KIR2DL4 regulation of the immune microenvironment in breast cancer, hopefully providing a rationale for combined HLA-G and immune checkpoints targeting for the effective treatment of breast cancer.

## Introduction

Breast cancer is the most common cancer diagnosed in women worldwide, with an estimated 2.3 million new cases and 0.7 million deaths in 2020 (1). Breast cancer is a very heterogeneous disease, both at molecular and histological levels. Five intrinsic subtypes of breast cancer were initially identified: Luminal-A, Luminal-B, HER2^+^, triple negative/basal like (TNBC) and normal like, which is based on the gene expression of estrogen receptor (ER), progesterone receptor (PR), and human epidermal growth factor receptor 2 (HER2) (2). The character of each subtype was associated with incidence, treatment response, rate of disease progression and metastasis. The conventional treatment options for patients with breast cancer include surgery, chemotherapy, radiotherapy, hormonal therapy and targeted therapy (3). Increasing evidence has showed that breast cancer microenvironment is not only composed of tumor cells but also of other different cell types, including endothelial cells, several stromal cell types, and immune cells (4). Due to the potential of the immune system in various new treatment strategies, immunotherapy has attracted the attention of researchers, including adoptive cell therapy, oncolytic virus and the most noteworthy immune checkpoint blockade therapy (5). Monoclonal antibodies against PD-1/PD-L1 and CTLA-4 have now entered clinical trials for the clinical treatment of triple negative breast cancer (6). Our recent study has showed that human leukocyte antigen-G (HLA-G) was a pivotal mediator of HER2 positive breast cancer resistance to trastuzumab and blockade of HLA-G can improve the antitumor activity of NK cells significantly (7).

HLA-G is a non-classical HLA Class I molecule that is first found specifically expressed in extravillous trophoblasts (EVTs) and plays a major regulatory role in maternal-fetal immune tolerance (8). During pregnancy, the fetus expresses paternal HLA, which are foreign antigens for maternal tissue, yet the fetus is neither rejected nor attacked by the maternal immune system (9). This phenomenon is due to the immune tolerance elicited by interaction between EVTs expressing HLA-G and leukocytes expressing inhibitory receptors (8). In 1998, Paul and his colleagues first reported the abnormal HLA-G expression in melanoma lesions rather than adjacent normal tissues (10). Accumulated evidence have showed that HLA-G is abnormally highly expressed in a variety of tumor cells and is involved in immune escape of tumors (11). All these findings suggest that HLA-G might be an important immune checkpoint in breast cancer immunotherapy.

Unlike the classical HLA Class I molecule, such as HLA-A, HLA-B, and HLA-C, the gene of HLA-G displays limited polymorphism (12). Seven different transcripts of *HLA-G* gene, namely HLA-G1 to G7, have been identified. HLA-G1 can be translated into both membrane-binding isoform and soluble isoform; HLA-G2, G3, and G4 transcripts can be translated into membrane-binding proteins; HLA-G5, G6, and G7 transcripts are templates for soluble proteins (11). Different HLA-G isoforms might underlie the diverse functions in cancer immunotherapy.

According to the recent studies, there are multiple receptors of HLA-G, including immunoglobulin-like transcript (ILT)-2, ILT-4, and killer inhibitory receptor (KIR) 2DL4, which are differentially expressed on immune cells (11, 13). ILT-2 and ILT-4 are the type I transmembrane glycoproteins with four extracellular immunoglobulin like domains, a transmembrane region, and an intracellular tail, which have four or three immunoreceptor tyrosine-based inhibitory motifs (ITIMs). ILT-2 and ILT-4 are expressed on T cells, natural killer cells (NK), and dendritic cells (DC) (14). Based on the structure of the intracellular tail, ILT-2/ILT-4 can initiate inhibitory signaling after binding to HLA-G, leading to immune suppression. KIR2DL4 is a member of the killer cell immunoglobulin (Ig)-like receptor (KIR) family, with two atypical extracellular domains, a positively charged arginine residue in the transmembrane region and an ITIM domain in its intracellular tail. The charged arginine residue of KIR2DL4 can recruit activation adaptors that contain immunoreceptor tyrosine-based activation motifs (ITAMs) (14). KIR2DL4 has both the activation and inhibitory signaling domains, suggesting that it can function as both activating and inhibitory receptor.

Our recent study found that KIR2DL4 synergizes with FcRγ to enhance NK cell activation and degranulation, while HLA-G binding to KIR2DL4 impairs the cytotoxicity of NK cells in HER2 positive breast cancer microenvironment (7). These findings suggested that KIR2DL4 might provide a switch for NK cell activity *via* its association with HLA-G. Increasing evidence has showed that HLA-G is involved in both innate and adaptive immune responses required for immune escape. With the unique structure, whether KIR2DL4 participates in cancer immunotherapy remains to be explored. In this review, we aim to summarize the roles of HLA-G/KIR2DL4 in breast cancer immune microenvironment.

## Molecular Structure of HLA-G and KIR2DL4

There are 8 exons and 7 introns in *HLA-G* gene. Most of the full-length HLA-G transcripts contains 7 exons, as exon 7 is usually removed by splicing. Compared with classical HLA Class I molecules, HLA-G is relatively short, the full length of which is only about 340 amino acids. The signal peptide was translated from exon 1; the extracellular α1, α2, and α3 domains were translated from exon 2-4, respectively; the transmembrane domain was generated by exon 5; and the intracellular cytoplasmic tail was generated by exon 6 (15). Accumulated evidences have shown that there are 7 isoforms of HLA-G. Each HLA-G isoform has its unique molecular structure due to different transcript splicing. HLA-G1 has both membrane-binding and soluble isoforms with extracellular α1, α2, and α3 domains, while HLA-G2/-G3/-G4 were membrane-binding isoforms. HLA-G2 has extracellular α1 and α3 domains; HLA-G3 has the only α1 domain; and HLA-G4 has α1 and α2 domains. HLA-G5/-G6/-G7 were soluble isoforms. HLA-G5 has extracellular α1, α2, and α3 domains; HLA-G6 has α1 and α3 domains; and HLA-G7 has the only α1 domains (11). The α1 and α2 domains contain the peptide-binding sites and the α3 domain can bind to β2-microglobulin (β_2_m) non-covalently. Recent studies have shown that a novel isoform of HLA-G without α1 domain can be detected in colorectal cancer patients and clear cell renal cell cancer patients, which might suggest distinct clinical significance (16, 17).

Killer cell immunoglobulin-like receptor 2DL4 (KIR2DL4) is the only KIR receptor recognized by HLA-G. KIR2DL4 belongs to the killer cell Ig-like receptors (KIR) family, which is expressed mainly on NK cells (7). KIRs can recognize both classical and nonclassical HLA Class I molecules. The *KIR* genes are located in chromosome 19q13.4, with each spanning 10,000-15,000 bp and separated by 1,000 bp. KIRs are encoded by up to nine exons: the leader peptide was encoded by the first two exons (exon 1 and exon 2), the two or three extracellular Ig-like domains (2D or 3D) by exons 3-5, the stem structure by exon 6, the transmembrane region by exon 7, and the cytoplasmic tail by exons 8-9 (18). Generally, KIRs have two or three extracellular immunoglobulin domains that were named KIR2D or KIR3D. Inhibitory KIRs including KIR2DL or KIR3DL contain a long cytoplasmic tail (L) with two immunoreceptor tyrosine-based inhibitory motifs (ITIMs). Activating KIRs like KIR2DS or KIR3DS contain a short cytoplasmic tail (S) and a positively charged residue in the transmembrane region, which can recruit adaptors that contain the immunoreceptor tyrosine-based activation motif (ITAM) (19). Unlike the conventional KIR2DL receptors, KIR2DL4 contains only one ITIM instead of two in its cytoplasmic tail and a positively charged arginine in its transmembrane region, which can recruit activation adaptors (19–21). Based on the unique structure, both activating and inhibitory functions have been described for KIR2DL4 on NK cells. Rajagopalan et al. found that KIR2DL4 is an activating receptor that can induce IFN-γ production by but not cytotoxicity of resting NK cells (22). Yusa and her colleagues showed that the single ITIM of KIR2DL4 can inhibit cytotoxic response of NK cells efficiently, which depends on SHP-2, but not SHP-1, and phosphorylated tyrosine (20). Our recent study has showed that HLA-G can desensitize breast cancer cells to trastuzumab by binding to the NK cell receptor KIR2DL4. Unless engaged by HLA-G, KIR2DL4 promotes ADCC function and forms a regulatory circuit with the IFN-γ production pathway, in which IFN-γ upregulates KIR2DL4 *via* JAK2/STAT1 signaling, and then KIR2DL4 synergizes with the CD16 to increase IFN-γ secretion by NK cells (7). Recent studies have showed that the IFN-γ production can impair the function of immune cells by upregulating PD-L1 on tumor cells (23–25). Consistent with previous reports, we observed that IFN-γ significantly increased the level of PD-L1 in breast cancer cells. Blockade of PD-L1 increased the cytotoxicity of NK cells against trastuzumab-treated HER2-overexpressing breast cancer cells (7). These findings suggested that since KIR2DL4 functions as either an activating receptor or an inhibitory receptor, it can affect the outcome of immunotherapy through the complicated cross-talk between different immune checkpoints and cytokines in the breast cancer immune microenvironment.

## HLA-G/KIR2DL4 Expression in Breast Cancer Immune Microenvironment

HLA-G expression in cancer microenvironment was regulated by several intracellular and extracellular mechanisms mediated by microRNAs, RNA-binding proteins, heat shock proteins, cytokines, et al. There are several microRNAs were correlated with the expression of HLA-G, such as miR-133a, miR-148a, miR-148b, miR-152, miR-199b-5p, miR-548q and miR-628-5p *et.al*, mainly investigated *in vitro* (26–28). Reches A et al. found that the RNA-binding protein, the heterogeneous nuclear ribonucleoprotein R (HNRNPR) can regulate the expression of HLA-G by binding to the 3’UTR of the HLA-G transcripts (29). Heat shock proteins was also reported to induce the HLA-G expression *via* the heat shock transcription factor 1 binding to the heat shock element of HLA-G promoter (30). The expression of HLA-G was regulated not only by intracellular post-transcriptional regulation, but also by extracellular mediators. HLA-G expression was found to be induced by progesterone *via* the interaction between progesterone receptor and progesterone response element (PRE) in the HLA-G promoter (31). Cytokines were also found to regulate the expression of HLA-G, such as GM-CSF, IL-10, TGF-β and IFN-β et al. (7, 32–34). All these findings indicated that the HLA-G expression in cancer microenvironment might be a key factor to investigate the progression of disease.

Many studies have shown that HLA-G/KIR2DL4 expression in immune microenvironment were correlated with the prognosis and progression of breast cancer. In 2002, Lefebvre et al. found that HLA-G was up-regulated at high frequencies in human breast cancer and it may impair anti-tumor immunity by recognizing inhibitory receptors, ILT-2 (35). Meanwhile, Urosevic and colleagues made an editorial comment, proposing that HLA-G and related killer cell inhibitory receptor might represent another mechanism for tumor immune escape (36). However, a recent study has showed that HLA-G expression did not significantly correlate with poor clinical outcome of cancer patients, which indicated that HLA-G expression might not necessarily participate in immunosuppression in carcinogenesis (37). Palmisano et al. analyzed the HLA-G expression at both RNA and protein levels in 25 breast cancer patient tissues. They failed to detect HLA-G expression in breast cancer tissues and cell lines, which was later found attributed to stromal cell contamination in tissue samples (38). In 2003, a comparative study reported that soluble HLA-G can be detected in the malignant ascites of breast cancer patients (39). In the same year, Korkola JE et al. Found in microarray assays that HLA-G were differentially expressed in lobular versus ductal breast cancer (40). Later investigations have established that HLA-G is an important marker of tumor immune escape (41, 42). Several studies have shown that the expression of HLA-G is significantly associated with progression and poor prognosis in a variety of tumors including aggressive breast carcinoma (43–45). Based on previous studies, whether HLA-G can be used as a marker for clinical diagnosis and treatment of breast cancer patients has also been extensively investigated (46, 47). In 2010, a comparative study reported that estradiol/progesterone-induced HLA-G expression can inhibit allo-cytotoxic lymphocyte response to human breast cancer MCF-7 cells (48). Chen et al. found that high soluble HLA-G levels was significantly correlated with the increased infiltration of Treg in breast cancer patients, which indicated that HLA-G might play an important role in the immunosuppressive breast cancer microenvironment (49, 50). HLA-G expression was also associated with subtypes of breast cancer. Provatopoulou X et al. found that HLA-G expression was increased in co-existing ductal and lobular breast cancer patients, compared to those with pure ductal cancer or pure lobular cancer alone (51). Yang’s team reported that there were more cases with high expression of HLA-G in non-luminal than in luminal subtypes, and HLA-G expression was associated negatively with the density of tumor-infiltrating lymphocytes (52). The mRNA stability of HLA-G was found associated with a 14-bp insertion/deletion in exon 8 of the 3’ untranslated region. A series of studies have shown that *HLA-G* polymorphism could be a diagnostic and prognostic marker for the susceptibility and pathogenesis of breast cancer in populations from different regions (53–59). MicroRNAs were also found to be involved in the regulation of HLA-G expression in breast cancer. Tao et al. found that G protein-coupled estrogen receptor (GPER) mediated the regulation of HLA-G by miR-148a, which was induced by estrogen (E2) in 2 human breast cancer cell lines, MCF-7 and MDA-MB-231 (60). It has been shown that HLA-G is expressed on immune cells such as NK cells and T cells (61, 62).

KIR2DL4 is the only killer cell Ig-like family receptor recognized by HLA-G. In 1999, Long EO et al. first reported that KIR2DL4, which is expressed at the surface of all NK cells including activated NK cells and resting NK cells, can bind to cells expressing HLA-G (22, 63). Goodridge et al. found that there are two KIR2DL4 alleles with either 9 or 10 consecutive adenines (9A or 10A) in exon 6, which encodes the transmembrane domain (64). The 9A alleles can produce a secreted receptor due to the splicing out of the transmembrane region, which might cause a lack of KIR2DL4 expression (65). In contrast, the “10A” alleles encode a membrane-expressed receptor that is constitutively expressed on resting CD56^bright^ and CD56^dim^ NK cells (65). The It has been described that the interaction of the KIR2DL4 receptor with the HLA-G molecule is mediated by the α1 domain, which indicated that all the seven identified isoforms of HLA-G can be recognized by KIR2DL4 (66). In breast cancer immune microenvironment, HLA-G can bind to its receptor, such as KIR2DL4, to induce immunosuppression. Ueshima C et al. found that human mast cells expressing KIR2DL4 can promote invasion of HLA-G-expressing malignant cells and the subsequent metastasis of breast cancer and choriocarcinoma (67). Recently, we have found that HLA-G expression can predict a low trastuzumab response in HER2 positive breast cancer patients (7). We also detected abundant KIR2DL4 expression on infiltrating NK cells in HER2 positive breast cancer tissues. These findings indicated that HLA-G/KIR2DL4 in immune microenvironment might play an important role in the progression of breast cancer and become a new target for breast cancer treatment.

## HLA-G/KIR2DL4 as the Novel Targets in Breast Cancer Immunotherapy

**Figure 1:**
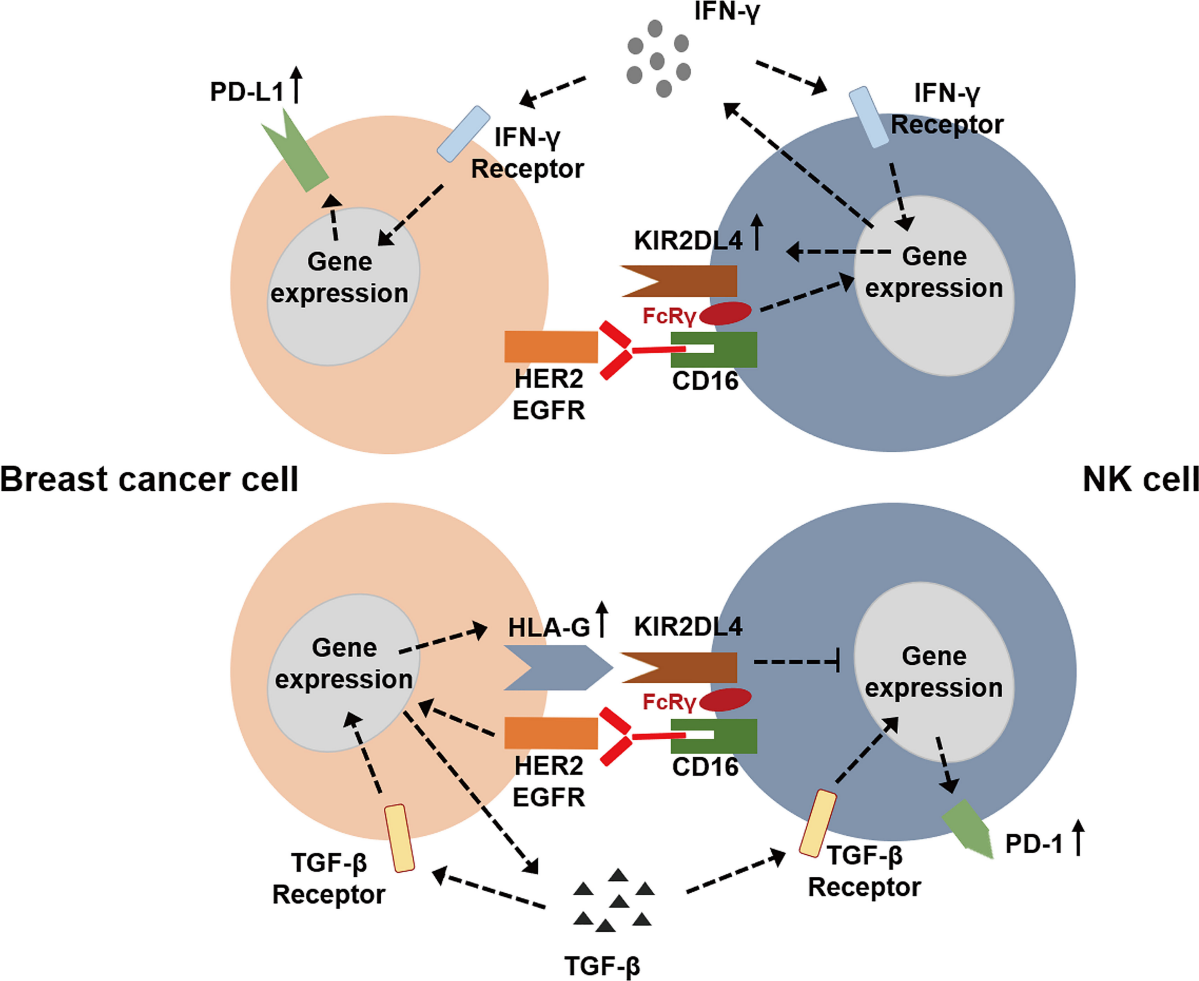
The potential roles of HLA-G/KIR2DL4 signaling in the NK cell mediated immunotherapy of breast cancer.

So far, the role of HLA-G/KIR2DL4 in breast cancer immunotherapy has been progressively elucidated. Roberti et al. found that blockade of ILT-2 with antibodies can restore cetuximab-mediated ADCC in triple-negative breast cancer patients and revert immunosuppression mediated by HLA-G (68). In 2016, Ishibashi and colleagues found that an MHC Class II-binding peptide HLA-G26-40 can elicit tumor-reactive CD4^+^ T cell responses effectively (69). With the wide application of immunotherapy in cancer, the relationship between immunological signature and progression of breast cancer has also been intensively investigated. A study reported that the expression of HLA-G was associated with both improved relapse-free survival (RFS) and overall survival (OS) of basal-like breast cancer, which might indicate a better status of lymphocyte infiltrating (70). Zhang’s team reported that overexpression of HLA-G in breast cancer cells was induced by abnormal DNA methylation modification, which was mediated by DNA methyltransferase (DNMT) and ten-eleven translocation (TET) (71). Therefore, TET inhibitor can prevent aberrant HLA-G expression *via* maintenance of DNA methylation, which provides a novel potential target for cancer immunotherapy (71). Jørgensen et al. also found that HLA-G expression was regulated partially by DNA methylation since the DNA methyltransferase inhibitor, 5-aza-2’-deoxycytidine, induced HLA-G expression, suggesting the feasibility of manipulating HLA-G expression for immunotherapy in breast cancer (72). In 2020, Schwich et al. reported that soluble HLA-G can induce a an immunosuppressive/exhausted phenotype, and the purified soluble HLA-G1 protein or extracellular vesicles derived from an HLA-G1-positive human breast cancer cell line, SUM149, can affect the anti-tumor function of CD8^+^ T cells by binding to ILT-2 (73). Our recent study reported that KIR2DL4, an alternative receptor of HLA-G, might be a novel target in breast cancer immunotherapy (7). We found that HLA-G impaired trastuzumab-triggered ADCC by binding to KIR2DL4 on NK cells, and blockade of HLA-G/KIR2DL4 can enhance the antitumor activity of trastuzumab *in vivo*. However, unless engaged by HLA-G, KIR2DL4 can promote ADCC and form a regulatory circuit with the IFN-γ production pathway *via* JAK2/STAT1 signaling in NK cells. In addition, paracrine TGF-β and IFN-γ in breast cancer microenvironment can induce PD-1/PD-L1 expression on NK cells and tumor cells, which might further enhance intercellular signaling that leads to immunosuppression. These findings demonstrated the applicability of combined HLA-G/KIR2DL4 and PD-1/PD-L1 targeting in the treatment of trastuzumab-resistant breast cancer (**Figure 1**).

## Conclusions

The immune system played an important role in the occurrence and progression of breast cancer. Many immunotherapy approaches have been investigated for breast cancer, including antibodies against tumor antigens, immune checkpoint blockade and CAR-T cells (4). Recent studies have shown that the combination of conventional chemotherapy or radiotherapy with immunotherapy contributes to improved outcome of breast cancer patients (74, 75). The therapeutic strategies for breast cancer have shifted from cytotoxic therapy to priming anti-tumor immune responses. Nonetheless, only a small part of breast cancer patients can currently benefit from immunotherapy. The relatively low responsive rate and the immunosuppressive tumor microenvironment even in the responding populations have limited the benefit of immunotherapy for breast cancer patients. The abnormal expression of immune checkpoint molecules is the main cause of immune escape. Thus, immune checkpoint blockade represents an important approach to reversing the immunosuppressive status of tumor microenvironment.

In this review, we summarized the special structural features of HLA-G and KIR2DL4. Then, we reviewed the expression of HLA-G/KIR2DL4 in breast cancer immune microenvironment and the potential of HLA-G/KIR2DL4 application in breast cancer immunotherapy. HLA-G, a non-classical HLA Class I molecule, was highly expressed in breast cancer tissues and associated with tumor progression and poor prognosis of patients. HLA-G engagement of its cognate receptors, ILT-2, ILT-4 and KIR2DL4 expressed on immune cells, can significantly induce immunosuppression and result in tumor immune escape. KIR2DL4 is the only KIR receptor that binds to HLA-G. KIR2DL4 contains an ITIM domain in its cytoplasmic tail and a positively charged arginine in its transmembrane region, which can recruit activation adaptors containing a ITAM domain. Based on its special structure, KIR2DL4 can mediate a complicated cross-talk between immune checkpoint and cytokines in breast cancer microenvironment, and dictate distinct outcome of immunotherapy depending on whether or not HLA-G is engaged. A deep understanding of the regulatory role of HLA-G/KIR2DL4 in the immune microenvironment of breast cancer might provide new ideas for the treatment of breast cancer.

## Author Contributions

Conceptualization, funding acquisition: A-GY. Writing original draft, review and editing: GZ and LJ. All authors contributed to the article and approved the submitted version.

## Funding

This work was supported by the National Natural Sciences Foundation of China (No. 81630069 and 81872303).

## Conflict of Interest

The authors declare that the research was conducted in the absence of any commercial or financial relationships that could be construed as a potential conflict of interest.

## Publisher’s Note

All claims expressed in this article are solely those of the authors and do not necessarily represent those of their affiliated organizations, or those of the publisher, the editors and the reviewers. Any product that may be evaluated in this article, or claim that may be made by its manufacturer, is not guaranteed or endorsed by the publisher.
